# The value of an embedded qualitative study in a trial of a second antidepressant for people who had not responded to one antidepressant: understanding the perspectives of patients and general practitioners

**DOI:** 10.1186/s12875-018-0877-4

**Published:** 2018-12-14

**Authors:** Carolyn A. Chew-Graham, Thomas Shepherd, Heather Burroughs, Katie Dixon, David Kessler

**Affiliations:** 1 Research Institute, Primary Care and Health Sciences, Keele University, Newcastle, Staffs ST5 5BG UK; 2 Centre for Academic Primary Care, Oakfield House, Oakfield Grove, Clifton, Bristol, BS8 2BN UK

**Keywords:** depression, Antidepressants, Primary care, Qualitative methods

## Abstract

**Background:**

Depression is the leading cause of disability worldwide, and is a major contributor to the overall global burden of disease. The number of prescriptions for antidepressants has risen dramatically in recent years yet up to 50% of patients who are treated for depression with antidepressants do not report feeling better as a result of treatment, and do not show the desired improvement on depression measures. We report a qualitative study embedded in a trial of second antidepressant for people who had not responded to one antidepressant, exploring the acceptability of a combination of antidepressants from the perspectives of both patients and practitioners, together with experiences of participating in a clinical trial.

**Methods:**

A qualitative study embedded in a randomized controlled trial investigating the effectiveness and cost-effectiveness of combining mirtazapine with Serotonin-Noradrenaline Reuptake Inhibitor (SNRI) or Selective Serotonin Reuptake Inhibitor (SSRI) antidepressants versus SNRI or SSRI therapy alone (the MIR trial). 59 interviews were conducted with people who declined to participate in the trial, people who completed the study and people who withdrew from the intervention, and 16 general practitioners.

**Results:**

Across the data-sets, four main themes were identified: the hard work of managing depression, uncertainties over the value of a second antidepressant, help-seeking at a point of crisis, and attainment and maintenance of a hard-won equilibrium.

**Conclusions:**

Exploring reasons for declining to participate in a trial of a second antidepressant in people who had not responded to one antidepressant suggests that people who are already taking one antidepressant may be reluctant to take a second, being wary of possible side-effects, but also being unconvinced of the logic behind such a combination. In addition, people describe being in a state of equilibrium and reluctant to make a change, reflecting that this equilibrium is ‘hard-won’ and they are unwilling to risk disturbing this. This makes some people reluctant to enrol in a clinical trial. Understanding a patient’s view on medication is important for GPs when discussing antidepressants.

**Trial registration:**

MIR Trial Registration: ISRCTN 06653773.

## Background

Depression is the leading cause of disability worldwide, and is a major contributor to the overall global burden of disease [1]. Currently, depression is thought to affect approximately one in ten of the population [2] and the prevalence of depressive symptoms may increase with age [3]^..^ Depression is more common in those people with long-term physical conditions [4].

GPs have been found to be good at recognizing moderate to severe depression [5], but they may be more likely to do so when patients present with psychosocial as opposed to somatic symptoms [6, 7]. A large WHO naturalistic study in 15 cities around the world (and in 11 languages), found that patients whose depression went unrecognized had milder depression at baseline and were not found to be at a disadvantage in terms of outcome [8]. Nevertheless a significant minority of people who might benefit from treatment remains undetected [9, 10].

People with depression are typically managed in primary care, and in the United Kingdom, depressive symptoms are the third most common reason to consult a general practitioner (GP) [11]. The National Institute for Health and Care Excellence (NICE) guideline on the treatment of depression [12] provides evidence-based guidance to primary and secondary care practitioners, in England, on the treatment of depression and promotes a stepped care model of treatment, recommending the least intensive therapies as a first line intervention. This is often a ‘talking treatment’ or psychological therapy [13] but referral to psychological therapists can be problematic due to waiting times for, and lack of availability of, appropriate and acceptable services [14]. The guideline suggests that where patients have moderate to severe, recurrent or chronic sub-threshold depression, antidepressants are indicated.

The number of prescriptions for antidepressants has risen dramatically in recent years; increasing by 7.2% (3.8 million items) between 2013 and 2014, in the NHS [15]. Indeed, antidepressants have shown a greater increase in the volume of prescribing in 2014 than drugs for any other therapeutic area, with over 57 million prescriptions being issued in England in 2014, at a cost of £265 million.

However, many patients do not respond to antidepressants. The STAR*D study (Sequenced Treatment Alternatives to Relieve Depression) found that half of those treated did not experience at least a 50% reduction in depressive symptoms following 12–14 weeks of treatment with a single antidepressant [16]. A substantial proportion of those who take their antidepressants in an adequate dose and for an adequate period, do not experience a clinically meaningful improvement in their depressive symptoms. This can be termed treatment resistant depression (TRD).

For patients who are deemed to have not responded to one antidepressant, the NICE guideline [12] suggests that General Practitioners (GPs) should re-consider the treatment option if there has been no response after 4 to 6 weeks of antidepressant monotherapy. The guideline describes a number of pharmacological strategies to attempt after inadequate response to initial treatments including combining antidepressants.

The ‘MIR’ trial evaluated the addition of mirtazapine to a Serotonin-Noradrenaline Reuptake Inhibitor (SNRI) or Selective Serotonin Reuptake Inhibitor (SSRI) antidepressant over placebo in primary care patients with TRD [17]. The embedded qualitative study, embedded in the MIR trial, reported here aimed to explore patients’ perspectives on being invited to participate in a trial [17] of a second antidepressant for TRD and the acceptability of combination drug treatments for depression to patients and GPs.

Patients’ health beliefs are central to reaching agreement about treatment between patient and practitioner, requiring a discussion which is inclusive of and considers the views and beliefs of both parties [18]. Furthermore, Patient beliefs and attitudes also influence adherence to antidepressant medication [19, 20]. Previous literature reporting patients’ views on the management of depression suggest that some patients prefer psychological treatments to medication, [21, 22] but these preferences vary depending on a number of factors including age, gender and their own understanding of depression [23, 24]. Negative views on antidepressants include concerns that they are addictive [25], and older people may be particularly reluctant to take antidepressants [26, 27].

It cannot be assumed that patients already on antidepressant medications will have either a positive attitude to, or a good understanding of, that treatment or will therefore be willing to take another antidepressant if their depressive symptoms remain. Evidence suggests that some patients using SSRI antidepressants would prefer to stop but are afraid to do so [20] and that when considering stopping the use of SSRIs, patients have a number of concerns about cessation which clinicians should attend to. [28, 29] In a meta-ethnographic synthesis of qualitative evidence it was found that patients were heavily engaged in negotiating a ‘medication career’ or ‘moral career’ [30] making choices about how to proceed with treatment and sense-making around their illness. This study also emphasizes the vital role that GPs play in facilitating patient decision-making, through discussion to aid understanding and meaning-making around depression and treatment experiences.

The views of patients who decline to take part in research can highlight salient attitudes about interventions. Barnes et al. [31] reported, in their qualitative study exploring views of people who had declined to participate in a trial of cognitive behavioural therapy (CBT) for Treatment Resistant Depression, that the acceptability of the trial intervention was an important factor in determining whether patients chose to participate in the trial.

O’Caithain, Thomas & Drabble et al. (2014) emphasises that the value of qualitative research to randomized controlled trials includes ‘improving the external validity of trials and facilitating interpretation of trial findings’ [32]. This paper reports a qualitative study embedded in the MIR trial, and aims to contribute to the understanding of TRD, as well as the acceptability of a combination of antidepressants from the perspectives of both patients and practitioners, with implications for how the intervention may be received more widely by patients and GPs in primary care.

## Methods

Ethical approval was obtained from the South East Wales Research Ethics Committee (12/WA/0353).

We conducted semi-structured interviews with people who were invited to participate in the MIR trial [17], but declined, trial participants (those who completed the trial, and who withdrew) to generate in-depth data on perspectives of participating in the trial, reasons for completion or withdrawal. In addition, we interviewed GPs to explore their experiences of participating in the MIR trial, perspectives on managing people with depression, with a focus on perspectives of antidepressant prescribing.

Participants were recruited to the MIR trial [17] from 106 general practices in Bristol, North Staffordshire and Shropshire, Hull, York and Exeter. A screening questionnaire was posted to 2299 potential participants. Respondents who declined to participate could give a reason on a form which provided closed questions to indicate their reason(s) for declining. There was also a free text response box for respondents to give additional details about or alternative reasons for declining. Respondents were also given an option to indicate their willingness to be contacted to be interviewed about their reasons for declining. The questionnaire was brief, to maximise the likely response. These data were collected throughout the recruitment period.

### Recruitment

If respondents indicated that they were willing to be contacted about their reasons for declining participation, this data was stored on a database, accessed by the trial team to invite a sample of willing respondents, categorized as ‘decliners’ to participate in a semi-structured interview. Sampling from within the ‘decliner’ population was purposive, on the basis of gender, age, geographic location and reasons for declining to participate, to gain maximum variation within the sample. Free text responses were also used to direct sampling when respondents had selected ‘other’ as a closed response and when details were given in the free text box which would indicate their suitability or unsuitability to be contacted about their reasons for declining. Once identified as suitable for contact, the research assistant (KD) made telephone contact with the respondent, gave details about the purpose of the call, the relationship of the qualitative study within the MIR trial and their rights as a potential participant of the study to confidentiality and to withdraw their consent at any time. They were then asked whether they had further questions and whether they would be willing to participate in a telephone interview. If verbal consent was given these interviews were usually conducted at the time of the initial phone call or arrangements were made to call the respondent at a more convenient time.

Sampling of trial participants was carried out at 12 weeks (once primary outcome measures assessed), taking gender, age and geographical location to gain maximum possible variation within the available sample. Participants were advised at the12 week assessment by the researcher that they may be contacted by study team to discuss their experiences of participation in the trial. The research assistant (KD) received regular reports from the trial teams on participants who had reached the primary outcome measure, and following review of the participant record, would attempt to contact participants, where the contact notes suggested it was appropriate to do so. Where there had been any serious or adverse events or where the participant had declined further contact after the primary outcome measure, these individuals were not invited to participate in the qualitative study. Participants were initially contacted by telephone and asked whether they would be willing to take part in an interview to discuss their experiences of being in the trial. If a trial participant agreed, an information sheet with further details of the study was sent to the participant and a provisional date for a face-to-face interview was made (usually 1–2 weeks after the date of the phone call to allow the information sheet time to arrive and the participant to consider the content).

When a participant withdrew from the trial, KD reviewed the participant record with the researcher or research nurse who had visited the participant, and contacted the ‘withdrawer’ to gain consent to an interview. If the person agreed, an information sheet was sent and a provisional date for a telephone interview was made. ‘Withdrawers’ were asked to send their consent forms back to the research team prior to the interview which was conducted by telephone.

GPs were recruited from the 106 practices participating with the MIR trial. Sampling of GP practices was purposive and selection based on the geographical location of the practice, gender and experience of the GP, to give maximum variation in the available population. Sampling was also contingent on whether the practice had patients who had been randomized to the MIR trial.

GPs and practice managers were initially contacted by email, with the information sheet as an attachment, inviting them to discuss their experiences of managing people with depression and views of using a combination of anti-depressants. If there was no response to the email, telephone calls were made to the practice. If a GP expressed interest in participating in an interview, the research assistant (KD) arranged a telephone interview at a time that was convenient to the GP.

### Data generation

Informed consent to participate in the qualitative study was obtained from all participants in the study. People invited to take part in interviews about their reasons for declining to participate in the MIR trial, were provided with a short questionnaire during the trial recruitment phase on which to indicate consent to be contacted about their reasons for non-participation. When these patients were contacted by telephone, they were asked to confirm that they had consented to taking part in a telephone interview and for their verbal consent to record the telephone interview. Participants were reminded about their rights as a participant to confidentiality and to withdraw their consent at the beginning and the end of the conversation respectively. This procedure for gaining consent to conduct and record telephone interviews was also followed when contacting GPs for telephone interviews for the study. Interviews with GP participants and decliners were conducted via telephone.

Patients participating in the trial were sent an information sheet in the post after their 12 week outcome measure had been taken by the researcher or research nurse, outlining information about participation in the qualitative study. For those participants who agreed to an interview, each participant was asked to sign a written consent form to indicate their consent to participate, and the researcher confirmed this consent further verbally before commencing the interview. The majority of interviews with patient participants were held face to face with the participant, in their own homes.

Data were generated using semi-structured interviews with participants conducted either via the telephone (patients who declined participation in the trial and general practitioners) or during face to face interviews (patients who completed or withdrew from the trial).

Topic guides for each group of participants (decliners, patients and GPs) were produced prior to the commencement of the first interviews and were amended and adapted in response to initial analysis of interviews and after discussion within the research team. The process was iterative throughout the period of data generation to better explore the concepts and themes which emerged as being important to the interviewees and required more in depth examination during subsequent interviews.

Data generated from each of these data sets were collected during each interview by the qualitative research assistant, using a digital audio recorder. Audio files were transcribed verbatim by an authorized transcription company and transcripts were stored securely by the research assistant in accordance with standard operating procedures. Each interview was checked against the audio-recording, cleaned and anonymized prior to analysis.

Data generation was continued until data saturation within each data-set was considered, by the research team, to have been achieved.

### Data analysis

Thematic analysis of the data [33] was conducted independently by authors CCG, HB and KD in the first instance. Subsequently the three members of the qualitative research team discussed their individual analysis and themes were agreed through discussion [33]. The three researchers were from different professional backgrounds (academic primary care, health services research and anthropology) which increases trustworthiness of analysis. [34]. Transcripts were read and re-read, themes arising during data generation and analysis within the team, and the topic guides modified iteratively as data collection and analysis progressed.

The qualitative research team met regularly to discuss and agree coding, revisiting the data from each dataset (and from analysis of other data-sets) in an iterative manner to verify coding and themes generated from these codes. This process was repeated until it was agreed that each individual data-set had reached saturation. Analysis was initially conducted within each data-set, then comparisons carried out across the datasets, in order to identify similarities and differences (disconfirmatory evidence). Discussion was continued until consensus was reached amongst the larger research team and PPIE group. NVIVO 10 [35] was used to store data and aid analysis.

The qualitative research team worked with a Patient and Public Involvement and Engagement (PPIE) group to discuss transcripts from the data-sets. The aim was to add value to the analysis with the lay partners adding value to the analysis by bringing their own perspectives and identifying further areas for the researcher to look for in the interviews [36]. Four meetings were held with the PPIE group over the course of data generation and analysis.

Members of the study PPIE group were recruited from members of the Keele Research User Group (RUG) which consists of over 100 members who contribute to all stages of development of studies. The study team invited people to participate in the study PPIE group if they had personal experience, or experience as a carer, of depression.

Two or three interview transcripts, or extracts of transcripts, were circulated prior to the PPIE meeting, and then the transcripts were discussed during the meeting, exploring what meaning the PPIE members gave to certain data extracts, and the research team offering their interpretations and checking this out with the PPIE group. The PPIE group members were supported by a RUG Advisor at each meeting.

## Results

We will present reasons given by people invited but who declined to participate in the MIR trial.

We will then describe the participants in the qualitative study, followed by key themes across the data-sets.

Reasons given for declining to participate in the MIR trial:

The reasons that people gave for not wanting to participate in the MIR trial are listed in Table 1.

**Table 1:** Reasons for declining participation in the MIR trial

	Reason	*N* = 4702 (25%)^a^
a	I do not want to take part in a research study	2298 (49%)
b	I plan to stop taking my current antidepressant	1822 (39%)
c	I do not want to take mirtazapine	1692 (36%)
d	I do not want to take a placebo	1328 (28%)
e	I only want to take one antidepressant	936 (20%)
f	I’m not taking antidepressants	818 (17%)
g	I am too busy	463 (10%)
h	I am not depressed	323 (7%)
i	Other reason(s)	1321 (28%)

^a^ Individuals were able to indicate more than one reason, thus percentages do not add up to 100%

The commonest reason for declining was not wanting to take part in a trial (49%). 36% of people invited indicated that they did not want to take mirtazapine; it is not known if this was because of prior experience of being prescribed this anti-depressant. Not wanting to take more than one antidepressant was given by 20% people invited to participate in the MIR trial. Interestingly, 17% of people suggested that they were not taking an antidepressant – even though they were being prescribed and SSRI or SNRI according to their GP records; 39% people said they planned to stop taking the current antidepressant.

### Qualitative study participants

A total of 59 interviews were conducted; 23 with decliners (Mean duration = 11 min 27 s) (Table 2); 23 completers and withdrawers (Mean duration = 47 min 35 s) (Table 3), and 14 with General Practitioners (mean duration 19 min 31 s) (Table 4).

**Table 2:** Decliners

Site	Participant total	Gender Male	Gender Female	Age Range (years)
Bristol	7	1	6	27–66
Exeter	6	2	4	40–54
Keele	6	3	3	32–76
Hull/York	4	2	2	35–68
Totals	**23**	**8**	**15**

**Table 3:** Completers and Withdrawers

Site	Participant total	Gender Male	Gender Female	Age range (years)
Bristol	11	5	6	32–63
Exeter	3	2	1	56–83
Keele	5	2	3	38–69
Hull/York	4	0	4	49–52
Total	**23**	**9**	**14**

**Table 4:** General Practitioners

Site	Participant total	Gender Male	Gender Female
Bristol	5	4	1
Exeter	5	4	1
Keele/ N Staffs/Shropshire	2	2	0
Hull/York	2	1	1
Total	**14**	**11**	**3**

#### Key themes across the data-sets

Key themes presented in this manuscript include ‘the hard work of managing depression’, uncertainties over the value of a second antidepressant, help-seeking at a point of crisis, and attainment and maintenance of a hard-won equilibrium.

Illustrative data will be presented with the gender and age of the patient participant given as identifiers in the case of trial participants (completers and withdrawers) and those who declined to participate (decliners). Data from GP transcripts will be identified with numbers reflecting the chronological order in which the interviews were conducted.

#### The hard work of managing depression

Respondents reflected on the history of their depressive symptoms, and there were rich descriptions of endeavours to manage their mental-health, including a broad range of self-management strategies and help-seeking.

Many respondents described delays in recognizing the cause of their depression, outlining repeated investigation for physical problems until a diagnosis of depression was achieved by default:

*“Erm, what it went back to was – I think it was postnatal depression, it started, and I didn’t realise what it was. Erm, that was [...] so that was 20 years ago and I thought I’d got ME or, or MS or something, because I, I, physically, couldn’t walk. I wasn’t particularly unhappy, but I, I was physically fatigued really, really fatigued. And I struggled with that for years, going for tests, and one thing and another, and it never came to anything, because it was depression.”*[Female, 55, decliner].

Once recognized as depression, respondents reported that they had tried numerous strategies and life-style changes to manage their own symptoms, including exercise and diet, seeking support from family and friends, taking steps to ease the pressure on themselves, and pursuing psychological and alternative therapies:

*“Well I’d been on websites to look at the condition….to look at what I could do, such as the omega-3, eating well, exercising, getting as much light as you can…all those sort of things.”* [Male, 57, decliner].

Respondents described the effort involved in ‘carrying on’:

*“you feel as if you don’t have as much confidence and you don’t feel erm what – erm like you don’t want to participate in things that you did want to participate in and you’re not really bothered about things. Obviously you are, but that’s how it makes you feel, you know. And also, like you haven’t got as much energy as you did have and you keep putting things off… ‘Oh yeah, I’ll do it tomorrow.’ And yet, I mean, I’m a very busy person. I have a full-time job and I work at least 40 h a week, so I mean I do hide it well, it’s on the inside I would say*.” [Female 59, completer].

All respondents indicated the hard work involved in trying to manage their symptoms and endeavouring to find something that might help:

*“...I’ve been doing a lot of meditation and, sort of, pursuing the mindfulness route […] I think to get out of depression, you have to probably put in a lot of different things in place to be able to tackle the things that have got you there, [okay] and exercise and eating well or trying to eat well even though I didn’t have an appetite.”* [Female, 37, decliner].

*“there’s a group called* (names third sector group*), to get in contact with them, and see what they would suggest, as well, and what they could do to help. And also, er, through work, I contacted, hmm, another similar group…and they directed me to, like, er, a series of one-to-ones or just, sort of, counselling, sort of soft counselling, or something [okay], just talking to somebody, talking therapy [uh-huh]. Er, a via* (a different third sector group*), I ended up doing a, erm, what was it called, which is more to do with, sort of, the food and overeating side of things, and I ended up having cognitive behavioural therapy with somebody, as well. Erm that was more along the line to control eating, which was, as opposed to dealing with depression in a particular way [okay]. But, yeah, I came away that day, from the doctor’s, with antidepressants.”* [Male, 55, completer].

Some respondents described an initial reluctance to take anti-depressants, but having got over this, suggested that they had come to terms with the need to use drugs to manage their mood. However, many respondents expressed concerns over the length of time that they would have to take their current antidepressant and were resistant taking a second antidepressant.

“*I felt […] that I didn’t wanna get involved in taking tablets for six, nine, 12 months. I’m already six months into taking them now [hmm], which is longer than I thought I would be…I thought, ‘Oh, I’ll get rid of it. I’ll be okay. I’ll have a few months or I’ll have a couple of months off. I’ll be back to my normal self,’ but it hasn’t worked like that..Erm, and whether another antidepressant would help I really don’t know.”* [Male, 55, decliner].

Such reluctance was sometimes due to a fear of (additional) side-effects:

***“****I didn’t want to start with the side effects what I got from the first one [uh-huh]. I didn’t want to start with new side effects and things.”* [Female, 4, decliner].

Whilst GPs described managing people with depression as ‘the bread and butter of general practice’, they reflected on the complexity of the work involved in the assessment of a patient:

*“I mean all of it is history taking initially, finding out a little bit about the, the, the physical problems, social problems and obviously their mental health problems. There might be more significant social connotations. So they might have alcohol related problems or drug use problems and things like that so that can make things a bit more challenging”* [GP04].

“*you can’t give a global answer to that actually because it’s incredibly tailored individual …to individual patient circumstances and patient beliefs […] so I’m - you know, I just think it’s - it’s just very individual I think we all adopt different consultation styles for different patients, don’t we?”* [GP02].

This multifaceted and individual assessment of patients who presented with depressive symptoms or ongoing depression, described by some GPs as a large component of their workload, was also described as one that can feel like a ‘battle’ and at times overwhelming:

*“Erm, so it’s, it’s inevitably a battle [yeah]. Well, not a battle, it’s a challenge to, to relate to the patient in the way that they understand. That’s what we do for a living, isn’t it? [Hmm] I suppose.”* [GP12].

In addition, GPs described the complexity in negotiating management of depression with patients:

*“erm I think it’s - some of it’s about previous response or lack of previous depression, really. [okay] Erm the, the - I think then that’s looking at the broader context of what the patient’s like, whether it’s a personality disorder erm and whether it’s err circumstantial, you know, whether it’s - there’s, there’s - it - there’s quite a few things that feed into that, really”* [GP10].

#### Maintaining equilibrium

The invitation to participate in a trial was described as posing a challenge, as managing depression was already seen as a series of complex decisions, with continued reflection and self-monitoring required. Respondents indicated that they were declining to take part because they feared disturbing the sense of balance they had achieved with their current antidepressant medication. The potential work involved in participation was felt to be a challenge to current stability:

***“****Because you know when you suddenly drop and you don’t know why? [Hmm] Erm, I’ve had a drop recently, so that, I don’t know what that was, but I had a drop recently, and I had to, sort of, look again at what I was doing [yeah]. Erm, so, adding something, changing something, is a bit scary for me [uh-huh] because I was feeling good, and I like feeling okay.”* [Female, 53, decliner].

“*but for me, taking something, erm, potentially taking something that could disrupt the way I am when I’m, you know, feel I’m on a steady level, I, I didn’t really want to, to take that risk.”* [Female, 27, completer].

Other respondents, whilst recognizing that something else may be necessary to help them manage their symptoms, expressed a preference for talking treatments:

*“….I’m on quite a low dose really, 20 milligrams of, erm, Citalopram, and I think it was doing the job it needed to do […] to get me to point where I could look at some issues.”* [Female, 39, decliner].

A number of interview participants who had declined to consent to participate in the trial stated that, had they been offered combination treatment at a point of crisis, when they felt out of balance and unable to cope, they might have agreed to take part in the trial because of their desperation to overcome their depression. In the following extract, the decliner participant is responding to a question from the researcher about whether they would have considered taking two antidepressants if they hadn’t felt better on one tablet. Their response suggests that if they had been ‘at rock bottom’ taking two tablets would not have been an issue if it offered the chance of getting better.

*“…I needed to do whatever it took to, you know, get well again, quite simply. So I, I would do whatever it takes. When you - I think when you really hit rock bottom you are prepared to do whatever it takes and I have absolute faith in my doctor*” [Female, 42, decliner].

One participant makes an extreme comparison, but makes the same point about being willing to accept whatever was necessary to escape being depressed.

“*I think, I think if you’d caught me when I was depressed, I would have taken anything to be honest, I would have done anything to get out of it […] in the first instance I struggled for a few weeks erm, thinking oh I can get out of this and it’s alright, it’s temporary and then it gets to a point where two, three weeks down the line, I just thought, I can’t deal with this, I’m on a sinking ship, so I think if the study had been then, and someone had offered me, you know, I don’t know, a shot of heroin a day to get out of it, I would have done it!*” [Female, 38, completer].

#### Why would another tablet help?

Some respondents expressed scepticism about the ‘chemical imbalance’ story that they felt had been told to them, to explain why a tablet would help their mood. Why a second anti-depressant would help if the first had not was questioned:

***(I)*** *: “…would you have had any concerns about taking two antidepressants?”*

***(P):*** “*Erm, no, I don’t think so…but I should wonder why I suppose I hadn’t had any, er, sort of beneficial reaction and maybe had to go to the doctors again and say it doesn’t seem to be working terribly well, what else can we do”* [Male, 76, decliner].

Respondents described the protracted sequence of trying out other antidepressants, increases in the dosage of their medication which had been either been ineffective or had worked for a limited time, then seemed to stop working, and referrals to talking therapies. Hoping that this trial would be another option for them to regain a sense of wellbeing was prevalent in accounts of these participants’ experiences:

*“I felt that the antidepressants, I’d already been on, you know, I was given 20 milligrams, and then they were increased to 40 [uh-huh], and yet I still wasn’t feeling, I wasn’t really feeling, er, 100% [uh-huh]. So I just felt that that [taking part in the trial] could be a different way of trying to get things, trying to get me a bit more better, you know”* [Male, 54, completer].

Participants in the trial were keen to get back to a level of coping and wellbeing they had experienced previously. They described that they were no longer feeling a beneficial effect from their medication and in some cases had sought out another option from their GP. The participant in the following extract gives an account of approaching her GP asking for them to ‘do something’:

*“I was taking sertraline erm probably from quite a while, really; about four months or so. And I felt they hadn’t made enough difference. [okay] Erm so I went back to the doctor and I was really worried and I said, ‘Look, I need some - either to up this medication or change it, do something”* [Female, 49, completer].

The opportunity to participate in the MIR trial came at a time when they were seeking an alternative option to help them manage their depression.

Patients who withdrew from the trial did so either because of side-effects of because they felt that they had regained their equilibrium:

*“I think the medication that I was on, got me, you know, on an acceptable level, you know. I’m not as bad as I was […] And then you get to a stage where yeah, things are acceptable. You just accept things and you just carry on”* [Female, 69, withdrawer].

“*Had my weight gain not been so severe I would’ve gone on longer taking the drug – and I told her* (the researcher) *that. I said to her that I couldn’t warrant having great thoughts and then dying of a cardiac arrest [laughs] because I’d put on so much weight. I have weight issues anyway, so it was just like the balance wasn’t right for me by the end of it”* [Female, 36, completer - withdrew after un blinding].

Although some GPs described a level of confidence in prescribing two antidepressants, all expressed caution about how long such a combination should be continued. Some speculated on how they might try to reduce one anti-depressant in a combination, but none of the GPs interviewed reported experience of stopping one in the combination:

*“I daresay we’d reduce one to zero, first of all, and see how it goes, and then reduce the other to zero [uh-huh]. I don’t think I would stop them both at the same time [yeah]. I don’t know [uh-huh], but I think I would, I’m sure I would do that, actually. I’d, I’d choose one and say, ‘Well, how are you doing?’ And, ‘We’re gonna have to start reducing these now [yeah], and I would suggest that,’ er, ‘we get you off one first of all, for a few weeks, and see how you feel, and then withdraw the other one slowly.”*[GP 12].

All GPs expressed concern about patients remaining on two anti-depressants, suggesting that patients were commonly prescribed SSRIs without a plan to withdraw:

*“it’s all right initiating it, isn’t it, and - but then I think then there becomes a bit more of a long-term issue about if you - how long do they stay on them both? [huh-huh] Do you - which one do you tail off first? [huh-huh] And erm you don’t - you wouldn’t necessary want people stuck on both antidepressants for time immemorial, which sometimes happens with SSRI, doesn’t it?*” [GP102].

Thus, GPs described a future dilemma if antidepressants were combined.

#### Help-seeking at a point of crisis

Those people who felt they were approached to participate in the MIR trial when they were at a point of crisis, described seeing the trial as an opportunity to deal with that crisis. Thus, participants detailed the current impact of their depression on their ability to cope and to the point where they recognized they needed help:

*“I had a lot of work stress going on as well [uh-huh], er, and it all got on top of me[…]. I was massively, er, overeating, erm, oversleeping, permanent low mood, just generally unwell […] So, er, at, sort of, that point, I went to the doctor and said, ‘Look, this is what’s going on. I need some help with this.’*”[Male, 52, completer].

Another patient reflects on how his feelings of desperation had led him to accepting referral into the trial by his GP, in the hope that it would offer alleviation of his current condition:

*“Well I think I was desperate enough to take her advice that it was more likely to be helpful than anything, than a hindrance, it wasn’t going to do any harm and it might do some good”* [Male, 83, completer].

GPs’ accounts reflected the perspectives of patients – that often help-seeking was at a point of crisis:

*“And, and if somebody’s depressed they really - if they’re, you know, if they’re at a watershed and they’ve actually come to the doctor’s about it […] because it’s often a crisis really when they’ve - or it’s been a huge step to actually come and do something about it.”* [GP102].

*“but a lot of the time, when people come and see us, er, it’s normally at the crisis point”* [GP08],

*“I think a lot of people by the time they’ve got to us they’re probably quite sort of… you know, er, they’re needing some help really.”* [GP03].

Such help-seeking at crisis point was not just described in new presentations, GPs described how patients with long-standing mood problems, would often re-present at a time of crisis:

*“Erm, clearly there’s a, quite an imperative to prescribe in some people, people who have been unwell for years. Some people want medication and don’t have the time or the inclination, or the enthusiasm to go for counselling, for a long period of time, and wait. They just want something done now, please.”* [GP114].

## Discussion

### Summary of findings

This qualitative study, embedded within a trial of a combination of two anti-depressants for people with treatment-resistant depression illustrates patients and GPs descriptions of the hard work involved in managing depression; patients described the need to maintain themselves at an equilibrium, and the importance of a ‘crisis point’ in the precipitation of help-seeking. People who declined to participate in the MIR trial [17] described feeling that they were at an equilibrium, and feared that participating in the trial would disturb that hard-won equilibrium. Experiencing a crisis-point seemed to be a motivator to make a change in management, and the offer of trying a second antidepressant within a trial could act as that motivator to change. Some ‘decliners’ expressed uncertainties about the role of medication in the management of depression (even though they were already taking one antidepressant) and feared that a second tablet would not help, or could not see the logic of a second medication.

Those people who participated in the trial (whether they completed or withdrew) described the persistent hope that something would help their mood, and this was something that the participation in the trial offered. The narratives of GP respondents reflected the patient data – describing the ‘hard work’ needed to manage people with depression, how ‘treatment resistance’ was about more than a poor response to an antidepressant, but rather about a complex patient presenting the GP a challenge in management. GPs described how patients often present at a crisis-point and how the MIR trial offered an option for them to help manage such patients.

### Comparison with previous literature

Qualitative research can address questions in trial recruitment that are not easily addressed by quantitative methods, by providing in-depth information on the experiences of participants and recruiters. [37] The commonest reason for people to decline to participate in the MIR trial [17] was not wanting to take part in a research study, as described in Barnes et al. qualitative study [31] nested in a trail of Cognitive Behavioural Therapy (CBT)for ‘treatment-resistant depression’. But, in contrast to Barnes’ qualitative study, where the ‘decliners’ expressed negative feelings about the treatment offered (CBT) in the trial, in our study ‘decliners’ who were interviewed, described how they were at an equilibrium which would be disturbed if they chose to participate in the trial, although a second antidepressant was not thought to be logical by a number of the ‘decliners’ interviewed. The patient may be perceived to be resistant to make a further change, having tried out various strategies and reached a position they were satisfied with, and feared losing. This illustrates the difficulty of the term ‘treatment resistant depression’ – the individual rather than the depression needs to be considered.

Hughes-Morley et al. [38] described how individuals declined to participate in a trial because they judged themselves ineligible or not in need of the trial therapy. Similarly, our analysis suggests that the ‘decliners’ we interviewed did not feel that they were in need of the trial intervention, a second antidepressant. MIR trial participants described experiencing a crisis as a motivator to participate in the trial, which resonates with Schofield et al. (2011) who reported that participants described their first course of antidepressants as typically occurring when they had ‘hit rock bottom’, having exhausted all other possibilities [39]. Schofield reported that participants described periods of experimentation where it was usual to stop and restart medication, often several times. Ultimately, these recurring cycles lead to participants becoming more expert about their condition and better able to make an informed decision about medication, yet still asking their GP for help. This description of patient expertise resonates with our analysis. Schofield also reported that for older people there was often an acceptance that their condition, and medication use, would be long term. [39] This acceptance might resonate with the concept of ‘equilibrium’ we describe in this study. In a meta-ethnography [30] Malpass and colleagues (2008) outline the role of the general practitioner in supporting decision-making about anti-depressant use, facilitating concordant relationships with patients regarding antidepressant use. Not having this discussion at entry into the trial may account for some people declining to participate. Malpass et al. [30] recommend that GPs are aware of the competing demands that patients experience at a decision-making juncture. We identified the importance of a ‘crisis’ in influencing a decision to try something new, or a sense of equilibrium in resisting change.

### Strengths and limitations

Exploring the reasons for non-participation in trials is still unusual (and difficult), and this study describes how such interviews can be incorporated into a trial recruitment procedure. A limitation of the study is that people who responded to the decliner questionnaire, and invitation to participate in a telephone interview about their reasons for declining to participate in the MIR trial, were a self-selected group, which may limit the generalisability of the findings. Perhaps people with stronger opinions about the trial might be more likely to respond. In addition, the telephone interviews were relatively short (mean duration less than 12 min). A strength of this study was involvement of the PPIE group in the analysis [36], although some challenges arose, including difficulties in recruiting a diverse range of members of the public to participate in the PPIE group, and the research team sometimes experienced difficulties keeping the PPIE members on track within meetings.

### Implications

Collecting information about those who decline to take part in research is a key way to learn about the acceptability of treatments being studied. In pilot studies for large-scale RCTs, gathering such information may be useful in refining recruitment estimates. Researchers recruiting to trials need to be sensitive to the patients’ prior experience of the same (or a similar) intervention being studied, their feelings about the intervention, and their views on their potential eligibility.

Similarly, when recruitment takes place with a primary care consultation, GPs need to explore patients’ reasons for declining to address any concerns or misconceptions about the research. This could increase recruitment to studies and thus ultimately contribute to increasing the evidence base.

Exploring reasons for declining to participate in a trial of a second antidepressant for depression suggests that people who are already taking one antidepressant may be reluctant to take a second, being wary of possible side-effects, but also being unconvinced of the logic behind such a combination. In addition, people describe being in a state of equilibrium and reluctant to make a change, reflecting that this equilibrium is ‘hard-won’ and they are unwilling to risk disturbing this.

The importance of achieving and maintaining an ‘equilibrium’ is a key finding of this qualitative study and should be incorporated into recruitment strategies in future trials of treatments for patients with depression not responding to an antidepressant, and for whom the addition of a second antidepressant, or switching an antidepressant, as suggested by the NICE guideline [12].

A crisis seemed to be the motivator for a patient with depression to try something new, including agreeing to take a combination of antidepressants, and this is the point at which people often consult their GP for help in routine practice. If a patient receives an invitation to participate in a trial when they are at such a point, they may be more willing to participate in the study. Whilst the MIR trial [17] evaluating the addition of mirtazapine to an SSRI, did not find convincing evidence of a clinically important benefit for mirtazapine in addition to an SSRI or SNRI antidepressant over placebo in primary care patients who were already on one antidepressant but remained depressed, it is likely that clinicians will continue to offer the addition of a second antidepressant, including mirtazapine, to patients with so called ‘treatment-resistant depression’. It is vital that the GP explores with the patient their views on antidepressants, what other strategies they have tried to manage their symptoms, and whether the patient feels they are at a point of crisis or equilibrium. This has implications for the education and training of GPs about the management of depression in primary care, and the importance of regular review and monitoring for people with depression.

## Conclusions

Understanding a patient’s view on medication is important for GPs when discussing antidepressants in the routine primary care consultation when a diagnosis of depression has been made and agreed. Understanding patients’ self-management strategies and what has precipitated their consultation is key to negotiating appropriate and acceptable management strategies.

## Abbreviations

CBT: Cognitive Behavioural Therapy
GP: General Practitioner
MIR trial: Mirtazapine added to SSRIs or SNRIs for treatment resistant depression in primary care: phase III randomised controlled trial
UK: United Kingdom
